# Astrocytes and the tumor microenvironment inflammatory state dictate the killing of glioblastoma cells by Smac mimetic compounds

**DOI:** 10.1038/s41419-024-06971-5

**Published:** 2024-08-15

**Authors:** Kyle Malone, Melanie Dugas, Nathalie Earl, Tommy Alain, Eric C. LaCasse, Shawn T. Beug

**Affiliations:** 1 Apoptosis Research Centre, Children’s Hospital of Eastern Ontario Research Institute, Ottawa, ON Canada; 2 Department of Biochemistry, Microbiology and Immunology, University of Ottawa, Ottawa, ON Canada; 3 Centre for Infection, Immunity and Inflammation, University of Ottawa, Ottawa, ON Canada

**Keywords:** Cancer microenvironment, CNS cancer

## Abstract

Smac mimetic compounds (SMCs) are small molecule drugs that sensitize cancer cells to TNF-α-induced cell death and have multiple immunostimulatory effects through alterations in NF-κB signaling. The combination of SMCs with immunotherapies has been reported to result in durable cures of up to 40% in syngeneic, orthotopic murine glioblastoma (GBM) models. Herein, we find that SMC resistance is not due to a cell-intrinsic mechanism of resistance. We thus evaluated the contribution of GBM and brain stromal components to identify parameters leading to SMC efficacy and resistance. The common physiological features of GBM tumors, such as hypoxia, hyaluronic acid, and glucose deprivation were found not to play a significant role in SMC efficacy. SMCs induced the death of microglia and macrophages, which are the major immune infiltrates in the tumor microenvironment. This death of microglia and macrophages then enhances the ability of SMCs to induce GBM cell death. Conversely, astrocytes promoted GBM cell growth and abrogated the ability of SMCs to induce death of GBM cells. The astrocyte-mediated resistance can be overcome in the presence of exogenous TNF-α. Overall, our results highlight that SMCs can induce death of microglia and macrophages, which then provides a source of death ligands for GBM cells, and that the targeting of astrocytes is a potential mechanism for overcoming SMC resistance for the treatment of GBM.

## Introduction

Glioblastoma (GBM) is the most common, aggressive and lethal glioma, comprising approximately 75% of new diagnoses and presenting a median overall survival of under 17 months [1]. Treatment involves maximal surgical resection, radiotherapy, and temozolomide chemotherapy [2, 3]. Recent addition of transdermal electric field radiation (antimitotic tumor-treating fields) has slightly improved progression-free and overall survival [4]. Nonetheless, central nervous system (CNS) tumors collectively present some of the highest mortality-to-incidence ratios of all cancers, with the highest treatment costs per patient [5–7]. Novel treatments are desperately needed for GBM.

GBM is characterized by high levels of proliferation, angiogenesis, infiltration into brain parenchyma, genomic instability, necrosis, intra- and inter-tumoral heterogeneity, and apoptotic resistance [8]. Among the myriad of apoptosis resistance factors involved in GBMs, the inhibitor of apoptosis (IAP) proteins represents a promising target for future therapies. The IAPs are defined by the presence of a baculovirus IAP repeat (BIR) domain, with many mammalian IAP family members possessing a really interesting new gene (RING) E3 ubiquitin ligase at the C-terminal end. The actions of these two domains allow the IAPs, most notably cellular IAP 1 and 2 (cIAP1 and cIAP2, encoded by the BIRC2 and BIRC3 genes, respectively) and X-linked IAP (XIAP, encoded by BIRC4), to suppress apoptosis through inhibition of caspases and by regulating NF-κB signal transduction through IAP RING E3 ubiquitin ligase activity [9]. In particular, cIAP2 has been identified as a key driver of gliomagenesis [10, 11] and poor prognosis [12, 13].

Many cancers resist intrinsic apoptotic cell death as a result of adaptations stemming from oncogenic driver mutations [14]. Notably, the IAPs are often overexpressed, conferring the ability to inhibit activation of obligate caspases [15]. Conversely, extrinsic cell death pathways are typically intact in cancer cells [14], including in all commonly used human GBM cell lines [16]. To that end, SMAC mimetic compounds (SMCs), which directly inhibit cIAP1, cIAP2 and XIAP, are currently being tested as therapeutics for solid cancers as well as lymphomas [17, 18]. SMCs demonstrate immunomodulatory effects via altered NF-κB signaling and by sensitizing cancer cells to inflammatory cytokine-induced cell death [19–28], circumventing impaired intrinsic apoptotic signaling. Notably, the effector functions of CD8^+^ T-cells are enhanced by SMCs, promoting tumor rejection [20, 29–31]. The use of SMCs in conjunction with immunotherapeutic strategies therefore provides a promising new avenue for improved GBM cell killing. Several groups have established that SMCs can sensitize GBM cells to temozolomide and radiotherapy [32–36]. In addition, our lab has shown that combination SMC and anti-programmed death-1 (α-PD-1) immune checkpoint blockade therapy produces durable cures in orthotopic murine GBM models [37]. Despite the remarkable efficacy of this combination approach, several tumors within each treatment cohort fail to respond in vivo regardless of the sensitivity found to in vitro SMC and TNFα treatment [37]. Here, we aim to assess the role of the CNS tumor microenvironment (TME) in affecting the efficacy of SMCs against GBM and determine what factors lead to the acquisition of associated resistance mechanisms to tumor cell death.

## Results

### Murine GBM cells are sensitive to SMC-induced cell death in a RIPK1-dependent manner

**Fig. 1:**
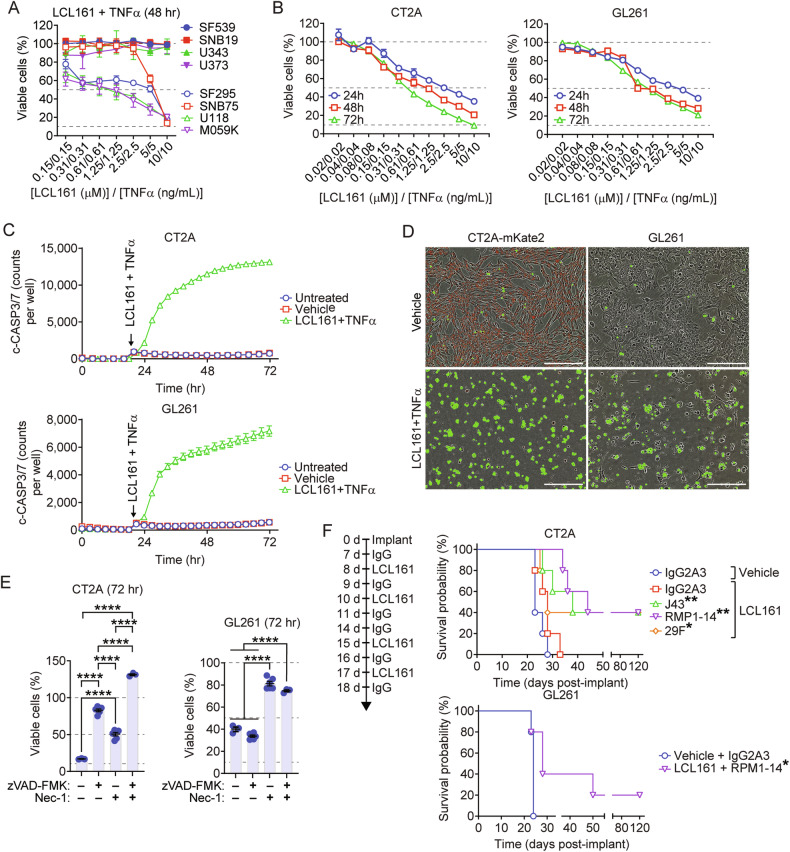
Murine GBM cells respond to SMC-mediated treatments in vitro and in vivo. **A** Human GBM cells were treated with a dilution series of combined LCL161 and TNF-α for 48 h. Viability was assessed by Alamar blue. *N* = 4 per treatment group. **B** CT2A and GL261 cells were treated with a dilution series of combined LCL161 and TNF-α for the indicated treatment time. Viability was assessed by Alamar blue. *N* = 3 per treatment group. **C**, **D** CT2A-mKate2 and GL261 cells were treated with vehicle or 10 µM LCL161 and 10 ng/mL TNF-α in the presence of the caspase-3/7 substrate DEVD488. Images were acquired via time-lapse microscopy and the number of DEVD488 was plotted. N = 3 per treatment group. Images in (**D**) are representative micrographs taken at 24 h. Scale bar: 300 µm. **E** CT2A and GL261 cells were treated with 10 µM LCL161 and 10 ng/mL TNF-α in the presence of caspase inhibitor zVAD-FMK (20 µM) and/or RIPK1 inhibitor necrostatin-1s (50 µM) for 72 h. Cell viability was assessed using Alamar blue. *N* = 3 for naïve or double-inhibitor treatment groups. *N* = 5 for single inhibitor treatments. ****P < 0.0001 by two-way ANOVA using Tukey’s HSD multiple comparison test. **F** Mice were implanted with 5 × 10^4^ CT2A or GL261 cells and treated orally with vehicle or 75 mg/kg LCL161 and intraperitoneally with 10 mg/kg of the indicated control or anti-PD-1 antibody as per indicated schedule. Data represent the Kaplain–Meier curve depicting mouse survival. Log-rank with Holm–Sidak multiple comparison. *N* = 5 per treatment group. *P < 0.05; **P < 0.01.

To reestablish the in vitro sensitivity of GBM cells to SMC treatments, human and mouse immortalized GBM cells were treated with a dilution series of the SMC LCL161 and TNF-α. Of the 8 tested human GBM lines, 4 were sensitive (SF295, SNB75, U118, and M059K) and 4 were resistant (SF539, SNB19, U3453, U373) to cotreatment over 48 h (Fig. 1A). Murine CT2A, GL261 and SMA-560 GBM cells were treated with differing concentrations of SMCs in the presence of TNFα. Monotherapy with either LCL161 or TNFα had no effect on viability (Supplementary Fig. 1), except at 50 μM in the case of LCL161 for CT2A and GL261 (a dose known to cause lytic cell death [38]). Combination of LCL161with TNF-α produced robust killing of both cell lines (Fig. 1B; Supplementary Fig. 2). Using the Incucyte caspase-3/7 DEVD488 dye, wherein the base substrate crosses cellular membranes and is cleaved to a fluorescent byproduct by activated caspase-3/7, we observed a substantial increase of fluorescence and, therefore, activated Caspase-3/7 via live cell imaging following treatment with the combination of LCL161 and TNF-α (Fig. 1C, D). To determine whether cell death mediated by combination LCL161 and TNF-α is via apoptotic or necroptotic pathways, we treated CT2A and GL261 cells with the combination and simultaneously inhibited obligate components of these pathways using the pan caspase inhibitor zVAD-FMK or the RIPK1 inhibitor Necrostatin-1s. After 72 h, both zVAD-FMK or Necrostatin-1s were able to inhibit LCL161 and TNF-α -induced cell death in CT2A, with the greatest effect observed following zVAD-FMK treatment (Fig. 1E). Only Necrostatin-1s protected against the combination treatment in GL261 cells. Therefore, CT2A requires caspases and RIPK1 for LCL161 and TNF-α-induced cell death and GL261 cells only require RIPK1, illustrating completely apoptotic (CT2A) and mixed apoptotic and necroptotic (GL261) responses.

Given these, the more inflammatory cell death prone GL261 cells [39] would be expected to respond better to immunotherapies. Immunostimulants increase serum, tumor and tumor-local proinflammatory cytokine levels [37, 40]. As SMCs induce inflammatory cell death in cells producing [41] or exposed to TNF-α (Fig. 1B) and themselves have broad immunostimulatory effects [20, 42, 43], combinations with immunotherapies such as oncolytic viruses [44] and immune checkpoint blockade in GBM [37] have produced potent synergistic effects. We evaluated the therapeutic efficacy of the combination of SMC with α-PD-1 in CT2A and GL261 intracranial syngeneic models as we have done previously [37]. In mice with CT2A tumors, combination LCL161 with three separate antibody isoforms of α-PD-1 (RMP1-14, J43, and 29 F) resulted in similar cure rates of ~40% (Fig. 1F, top), consistent with previous results [37]. RMP1-14 was chosen for all further in vivo work involving α-PD-1 blockade. We were also able to obtain long-term cures in the GL261 model (20% survival; Fig. 1F, bottom).

### Extracellular matrix proteins and hypoxia moderately protect against SMC and TNF-α induced GBM cell death

**Fig. 2:**
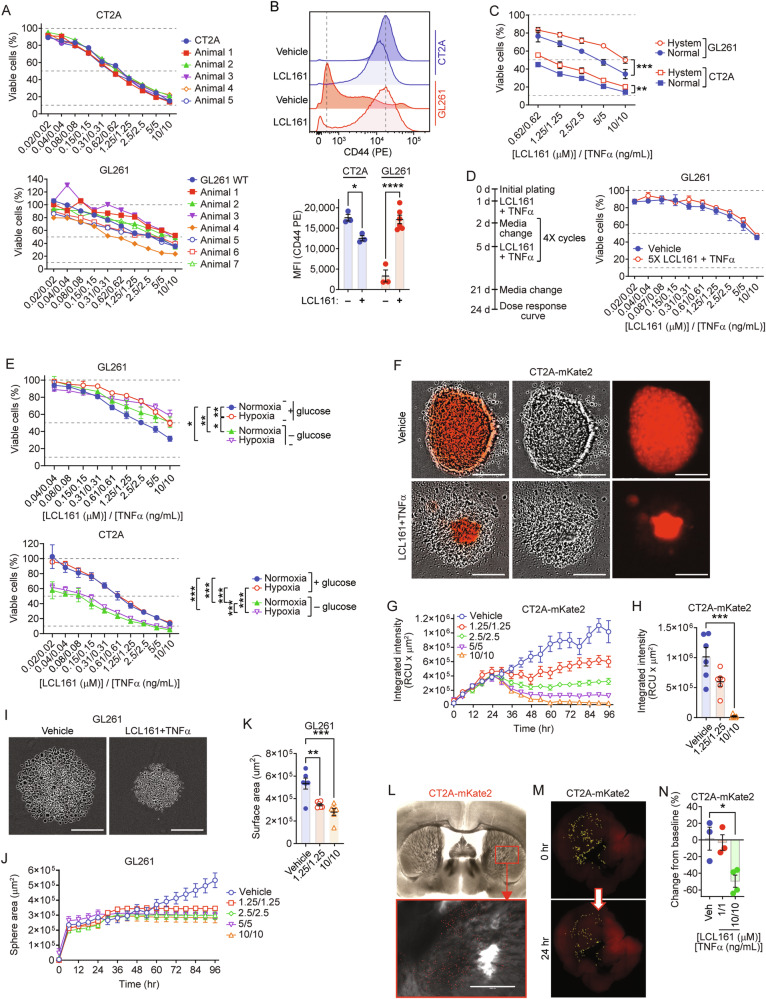
Hypoxia and extracellular matrix proteins are not significant factors mediating SMC-induced cell death. **A** CT2A and GL261 cells from LCL161 and anti-PD-1 resistant tumors were treated in vitro with the indicated dilution series of LCL161 and TNF-α for 24 h. Viability was assessed by Alamar blue. *N* = 3 per group. **B** Flow cytometric analysis of CD44 (PE) expression on CT2A and GL261 cells treated with 10 doses of 10 μM LCL161. Bar plots are mean fluorescent intensity (MFI) or CD44 expression. N = 3 for all CT2A and GL261 naïve groups; *N* = 6 for GL261 LCL161 treated. *P < 0.05; ****P < 0.0001 by two-way ANOVA using Tukey’s HSD multiple comparison test. **C** Alamar blue viability assays of CT2A and GL261 cells cultured on Hystem extracellular matrix gel and treated with dilution series of LCL161 and TNF-α for 24 h. *N* = 3 per treatment. **D** GL261 cells were treated five times with 10 µM LCL161 and 10 ng/mL TNF-α for 24 h per treatment as per indicated schedule and viability was assessed via Alamar blue. **E** Alamar blue viability of GL261 and CT2A cells treated with a dilution series of LCL161 and TNF-α under hypoxia and/or no glucose conditions. *N* = 6 for normoxia complete glucose. *N* = 3 for remaining treatment groups. *P < 0.05; **P < 0.01; ***P < 0.001 by two-way ANOVA using Tukey’s HSD multiple comparison test. **F**–**K** CT2A-mKate and GL261 cells were cultured as spheres and treated with the indicated dilution series of LCL161 and TNF-α at 24 h in culture. Fluorescence intensity (CT2A-mKate2) and size (GL261) were measured using time-lapse imaging and plotted at 72 h post treatment. *N* = 6 per treatment group. Scale bar: 800 µm. **L** Mouse brains were sectioned into 250 µm slices and 48 h later implanted with 5 × 10^3^ CT2A-mKate2 cells in the left striatum. Scale bar: 1 mm M-N) CT2A-mKate2 cells were enumerated before and 24 h after 10 μM LCL161 and 10 ng/mL TNF-α treatment (**M**) or of the indicated concentrations of LCL161 and TNF-α (N). *N* = 3 for vehicle and 1/1 treatment groups; *N* = 4 for 10/10 treatment. *P < 0.05; **P < 0.01; ***P < 0.001; ****P < 0.0001 by one-way ANOVA using Tukey’s HSD multiple comparison test.

As SMCs are only partially effective in curing intracranial gliomas, we ascertained whether treatment resistance is a tumor cell intrinsic or extrinsic response to identify resistance mechanisms and potential novel rational combinations to enhance therapeutic efficacy. We first evaluated whether CT2A and GL261 cells implanted within the brain acquired intrinsic cellular resistance mechanisms. We isolated tumor cells from mice treated with SMC and α-PD-1 that reached endpoint and evaluated for their responsiveness to LCL161 and TNF-α treatment ex vivo. In all cases we observed that isolated tumor cells remained sensitive to LCL161 and TNF-α cotreatment in a dose-dependent fashion (Fig. 2A). We next tested whether SMC response or resistance is affected by modifying in vitro culture conditions to recapitulate elements from the in vivo setting. We assessed the impact of hyaluronic acid (HA, a major component of the brain extracellular matrix (ECM)) on SMC sensitivity. Both CT2A and GL261 cells express CD44, the ligand for HA (Fig. 2B). Daily treatment over 10 days with 10 µM of LCL161 led to minimal reduction of CD44 expression in CT2A cells but conversely significantly increased CD44 expression in GL261 cells (Fig. 2B). These GL261 results are consistent with findings showing human glioma stem cells treated with the SMC birinapant for 7 days lead to increased expression of CD44 [45]. Sensitivity to LCL161 and TNF-α cotreatment is reduced in both CT2A and GL261 cells when cultured on an HA-containing ECM protein (Fig. 2C). To assess whether repeat SMC and TNF-α treatment leads to the development of resistant populations, we subjected GL261 cells to high dose LCL161 (10 µM) and TNF-α (10 ng/mL) for 5 treatment cycles as outlined in Fig. 2D. We observed that after this regimen, cells remain sensitive to subsequent treatments (Fig. 2E), suggesting sensitivity is maintained regardless of treatment number. Hypoxia contributes to GBM aggression [46, 47] and immunotherapy resistance [48]. In GL261 cells, both lack of glucose and low oxygen levels offered moderate protection against high dose LCL161 and TNF-α (Fig. 2E). Conversely, hypoxia had no significant impact on CT2A cells, while lack of glucose significantly reduced viability.

When cultured under spheroid conditions, both mKate-2 tagged CT2A or GL261 cells remained sensitive to LCL161 and TNF-α (Fig. 2F–K), suggesting the three-dimensional nature of in vivo tumors is not a major resistance factor. Together, these results indicate that either transient signals from the tumor microenvironment (TME) confer resistance to SMC-mediated cell death, or that murine GBM cells remain sensitive in vivo. To determine whether unique features of the CNS itself could transiently induce resistance, fluorescent CT2A-mKate2 cells were grown on top of organotypic brain slices in culture. Sensitivity to medium doses of LCL161 and TNF-α was abolished, although CT2A-mKate2 cells were still sensitive to high doses of the cotreatment (Fig. 2L–N, Supplementary Fig. 3). These results indicate that extrinsic cell death pathways engaged by simultaneous IAP blockade and TNF-α signaling remain intact within the CNS milieu contingent upon sufficient SMC and TNF-α reaching GBM cells. Optimizing drug delivery methods and maximizing intratumoral inflammation therefore represent avenues of enhancing SMC-based anti-GBM immunotherapies. As the TAMM and astrocyte populations play key roles in neuroinflammation, GBM biology and resistance to immunotherapies, we next aimed to examine effects of SMCs on these cell types.

### SMCs induce macrophage and microglia cell death

**Fig. 3:**
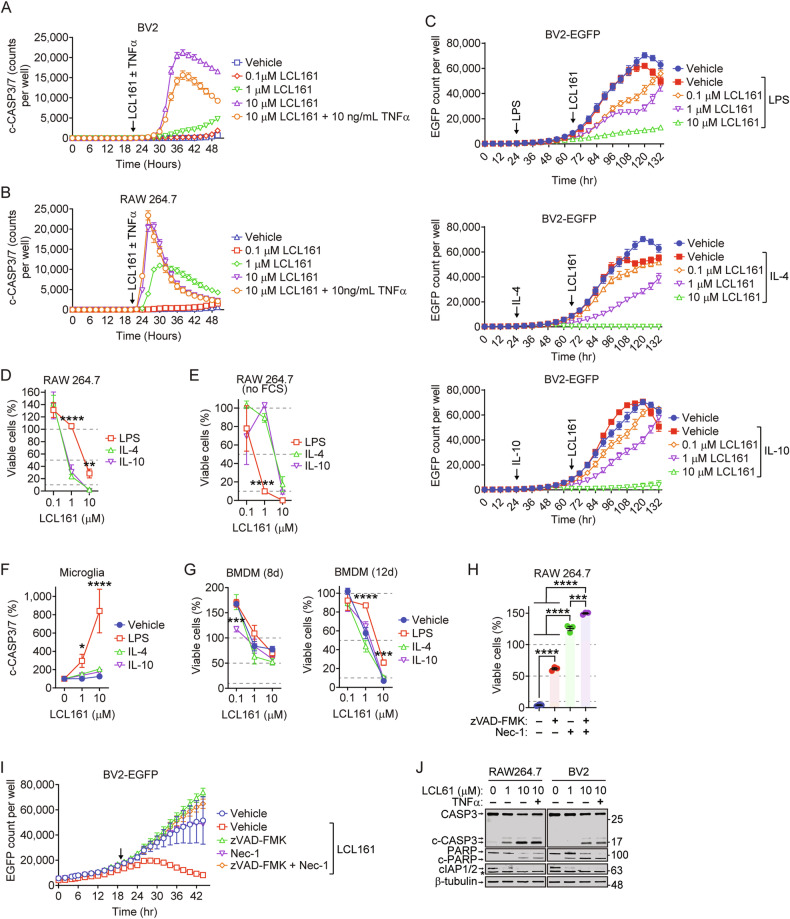
SMC treatment induces the death of microglia and macrophages. **A**, **B** BV2 and RAW cells were treated with indicated doses of LCL161 and TNF-α and then assayed for activated caspase-3/7 using live imaging. Arrows denote time of treatment. *N* = 6 per treatment group. **C** BV2-EGFP cells were treated for 24 h with LPS (100 ng/mL), IL-4 (20 ng/mL) or IL-10 (20 ng/mL) and subsequently treated with indicated concentrations of LCL161. EGFP-positive events were quantified over time via live microscopy imaging. Shaded area represents pre-treatment time with indicated cytokines. End of shaded area represents introduction of LCL161. *N* = 3 per treatment group. **D**, **E** RAW cells were treated as in (**C**) with subsequent introduction of indicated doses of LCL161 in the presence (**D**) or absence (**E**) of FCS. Viability was assessed using Alamar blue. *N* = 3 per treatment group. **F** Primary mouse microglia were treated as in (**C**) and then treated with indicated doses of LCL161 Activated caspase-3/7 was quantified using live imaging at 24 h post treatment. *N* = 3 per treatment group. **G** Bone marrow progenitors isolated from femurs were differentiated into macrophages for 8 or 12 days. Subsequently, cells were treated as in (**C**) and subsequently treated with indicated doses of LCL161. Viability was assessed using Alamar blue at 24 h. *N* = 3 per treatment group. *P < 0.05; **P < 0.01; ***P < 0.001 by two-way ANOVA using Tukey’s HSD multiple comparison test. **H**, **I** RAW and BV2-EGFP cells were treated with 10 µM LCL161 in the presence of zVAD-FMK (20 µM) and/or necrostatin-1 (50 µM) for 24 h. Viability was assessed by Alamar blue (**H**) or EGFP counts (**I**; arrow indicates treatment point). *N* = 3 per treatment group. ***P < 0.001; ****P < 0.0001 by one-way ANOVA using Tukey’s HSD multiple comparison test. **J** Western blot illustrating cIAP1/2 degradation and caspase-3 and PARP cleavage in response to LCL161 treatment.

Macrophages and microglia represent the most common infiltrating immune population in GBM [49], playing fundamental roles in gliomagenesis and immunosuppression [50]. Previous work from our lab has shown that SMC and α-PD-1 antibodies reduce the proportion of myeloid-derived suppressor cells within GBMs [37], attributed to the effect of α -PD-1 treatment. Alterations in polarization states of TAMMs and microglia in response to SMCs have not been thoroughly explored in a GBM context. Preliminary work was undertaken in vitro using the immortalized murine microglia cell line BV2 and macrophage cell line RAW264.7 (hereafter referred to as RAW). We assessed for the effects of SMCs on phagocytic potential of these cells, of which M2-polarized populations show higher phagocytic activity [51]. High-dose LCL161 treatment abolished RAW and BV2 phagocytic capacity (Supplementary Fig. 4), coinciding with dose-dependent caspase-3/7 activation in both cell types (Fig. 3A, B), illustrating LCL161 induces RAW and BV2 cell death.

There remains the possibility that immunosuppressive cytokines may affect sensitivity to SMC-mediated death of microglia and macrophages. Pre-treatment with LPS, IL-4 or IL-10, did not affect response to high-dose LCL161 treatment in either EGFP-tagged BV2 microglia (Fig.3C, assessed via measuring EGFP count) or RAW macrophages (Fig. 3D, assessed using Alamar blue viability assay). Serum-containing cultures have been found to inherently affect the cytokine secretion profile and differentiation capacity of mesenchymal stem cells [52]. Under FCS-free conditions, treatment with IL-4 and IL-10 protected RAW cells from 1 µM of LCL161. This resistance is lost at 10 µM (Fig. 3E). BV2 cell viability was significantly reduced under FCS-free conditions (Supplementary Fig. 5). We next assessed whether this sensitivity to SMC is evident in primary microglia and macrophages. Naïve and IL-4/IL-10 treated microglia did not display significant increases in cleaved caspase-3/7 following LC L161 treatment. Significant increases were observed in LPS-treated cells (Fig. 3F). On the other hand, 10 µM LCL161 treatment lead to death of murine bone marrow-derived macrophages (differentiation confirmed in Supplementary Fig. 6) regardless of pre-treatment or days in differentiation conditions (Fig. 3G). Treatment of RAW macrophages or BV2 microglia with 10 µM LCL161 in the presence of zVAD-FMK or Necrostatin-1 confirmed cell death occurs in a RIPK1- and caspase-dependent fashion (Fig. 3H, I), with cIAP1/2 degradation and caspase-3 and PARP cleavage confirmed via western blotting (Fig. 3J). These results indicate that microglia and macrophages are sensitive to high doses of SMC and that the eradication of these populations may be a mechanism for SMC efficacy in vivo.

### Hypoxia and TGFβ protect macrophages and microglia against the cytotoxic effects of SMCs

**Fig. 4:**
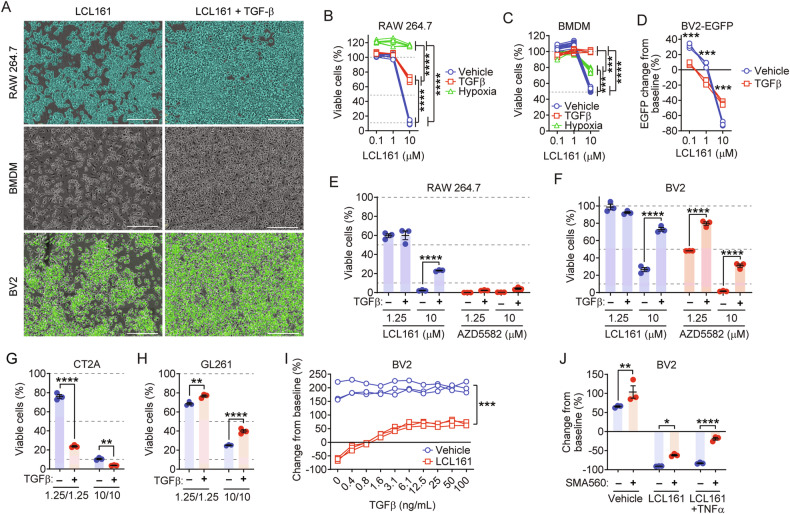
Hypoxia and TGFβ protect microglia and macrophages from LCL161-induced cytotoxicity. **A**–**F** RAW (*N* = 6 per treatment group), BMDM (*N* = 5 for TGF treated, *N* = 12 for other treatment groups) and BV2 (*N* = 3 per treatment group) cells were treated with 20 ng/mL TGFβ or cultured in 5% O_2_ for 24 h and then treated with DMSO or the indicated doses of LCL161 or AZD5582 (*N* = 3 per treatment group) for 24 h. Cell viability was assessed using Alamar blue. Change from baseline was calculated using BV2-EGFP counts immediately before and 24 h post treatment. Scale bar: 300 µm. **G**, **H** Cells were pre-treated with TGFβ treated as in (**A**). Viability of CT2A and GL261 cells in response to subsequent treatment for 24 h with 10 µM LCL161 and 10 ng/mL TNF-α was assessed by Alamar blue. *N* = 3 per treatment group. **I** BV2-EGFP cells were treated with varying concentrations of TGFβ for 24 h and then treated with 10 µM LCL161 for 24 h. The number of BV2-EGFP cells was enumerated by counting EGFP-positive events via live imaging, and the percent change in counts from prior to LCL161 treatment was calculated and plotted. *N* = 3 per treatment group. **J** BV2-EGFP cells were co-cultured with SMA-560 cells and treated with the combination of 10 μM LCL161 and 10 ng/mL TNF-α for 24 h. EGFP events were enumerated by live cell imaging. *N* = 3 per treatment group. *P < 0.05; **P < 0.01; ***P < 0.001; ****P < 0.0001 by two-way ANOVA using Tukey’s HSD multiple comparison test.

We evaluated the effect of alternative sources of GBM-mediated drug resistance and immunosuppression. Transforming growth factor-β (TGFβ) is a key prognostic cytokine in the GBM TME, with multifaceted roles in glioma stem cell maintenance and immunosuppression [53]. TGFβ and hypoxia both significantly reduce LCL161-mediated death of RAW, BMDM and BV2 cultures (Fig. 4A–D). TGFβ has been found to increase levels of IAPs, specifically XIAP [54]. We therefore compared the ability of the dimeric SMC AZD5582, which is more potent for antagonizing the IAPs compared to the monomeric LCL161 [55, 56], in the killing of TGFβ-treated RAW and BV2 cultures. AZD5582 treatment induced complete loss of RAW viability despite the presence of TGFβ (Fig. 4E). In contrast, TGFβ treatment still rescued AZD5582-mediated death of BV2 cells (Fig. 4F), although significantly less so than observed in LCL161 treated cultures.

We next assessed whether the protective effect of TGFβ also applied to GBM cells. TGFβ significantly enhanced the cytotoxicity of LCL161 and TNF-α cotreatment in CT2A cells (Fig. 4G). In contrast, TGFβ treatment conferred an increase in GL261 survival (Fig. 4H), although this was minor compared to those previously discussed in macrophages and microglia. We then evaluated for the minimum concentration of TGFβ required for this protective effect. Treatment with TGFβ alone did not alter BV2 growth rate, regardless of concentration. A significant protective effect against 10 µM LCL161 was observed beginning at 0.8 ng/mL (Fig. 4I). To determine whether GBM-secreted TGFβ would be sufficient to confer resistance to high dose LCL161, BV2-GFP microglia were co-cultured with SMA-560 murine GBM cells characterized by TGFβ secretion [57]. The presence of SMA-560 cells increased growth of BV2 microglia and conferred slight protections against LCL161 (Fig. 4J). TGFβ therefore represents a targetable macrophage and microglial survival factor against LCL161 treatment.

### Astrocytes enhance SMC-induced death of GBM cells

Astrocytes represent the largest cell population within the CNS, playing key roles in neuroinflammation, adopting reactive phenotypes similar to monocytes depending on the inflammatory context (A1: reactive, inflammatory; A2: alternative, anti-inflammatory) [58]. Various astrocyte-derived cytokines and growth factors enhance GBM migration and survival[59, 60], with media from astrocyte-GBM co-cultures increasing the expression of anti-apoptotic BCL-2 family proteins in naïve GBM cells [60]. The impact of IAP blockade and immunomodulation on astrocyte-GBM cell interactions has yet to be explored.

**Fig. 5:**
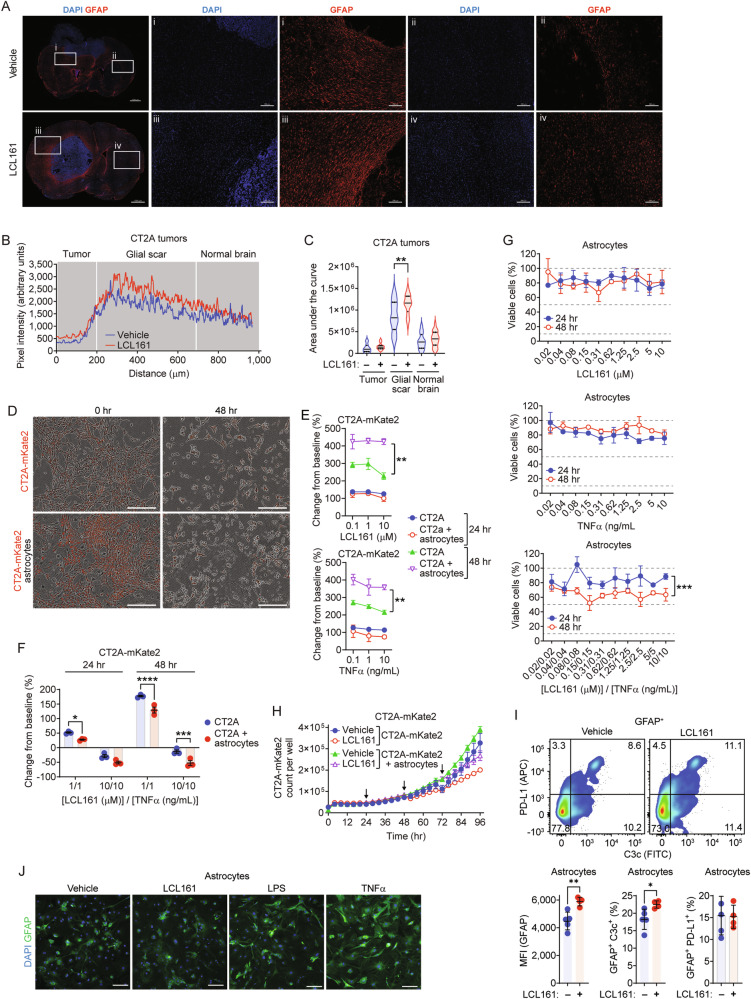
SMCs enhance astrocyte reactivity to promote GBM cell death under inflammatory conditions. **A**–**C** Mice with intracranial GL261 tumors were treated orally twice with vehicle or 100 mg/kg LCL161. At day 18 post-implant, brains were processed for immunohistochemical expression of GFAP surrounding the tumor (i, iii) or outward on ipsilateral side (ii, iv). GFAP intensity was calculated at the tumor border and plotted as mean (**B**) and area under the curve (**C**). *N* = 3 vehicle, *N* = 5 LCL161 treated animals per group. Scale bar: 1 mm or 200 µm as indicated. **D**–**F** Mouse cortical astrocytes were co-cultured with CT2A-mKate2 cells and treated with LCL161, TNF-α or the combination. Viable cells were enumerated by tracking mKate2-positive events using live cell imaging. Scale bar: 300 µm. N = 3 per treatment group. **G** Astrocytes were treated with dilution series of LCL161, TNF-α or the combination for 48 h. Viability was assessed using Alamar blue. N = 3 per treatment group. **P < 0.01; ***P < 0.001; ****P < 0.0001 by two-way ANOVA using Tukey’s HSD multiple comparison test. **H** Astrocyte and CT2A-mKate2 co-cultures treated with three daily doses of 10 µM LCL161 (arrows) and the number of mKate2-positive events was plotted over time. *N* = 3 per treatment group. **I** Flow cytometric analysis of GFAP+ mouse cortical astrocytes treated with 10 µM LCL161. Cells were analyzed for MFI of GFAP (PE-CF594) and proportional C3c (FITC) and PD-L1 (APC) expression. *N* = 5 per treatment group. *P < 0.05; **P < 0.01 assessed by *t*-test. **J** Mouse cortical astrocytes were treated with LCL161 (10 µM), LPS (500 ng/mL) or TNF-α (10 ng/mL) for 24 h and assayed for GFAP expression via immunocytochemistry. Scale bar: 200 µm.

We evaluated the effects of SMC treatment on astrocyte reactivity in the GL261 mouse model of GBM. LCL161 treatment significantly increased the expression of glial fibrillary acidic protein (GFAP), a major marker of reactive astrocytes [61], within the tumor border with no significant impact on expression distally (Fig. 5A–C), illustrating a greater tumor-local astrocyte involvement following SMC treatment.

We next undertook co-culture assays of GBM cells with astrocytes to assess their role in GBM responses to SMCs. The growth rate of CT2A cells treated with LCL161 or TNF-α alone was significantly greater in co-cultures than matched monocultures (Fig. 5E, Supplementary Fig. 7), suggesting astrocytes provide supportive factors promoting CT2A growth. However, we observed a significant decrease of viable CT2A cells under co-culture conditions when treated with LCL161 and TNF-α (Fig. 5F). Treatment with LCL161, TNF-α or the combination had no significant, dose-dependent impact on astrocyte viability (Fig. 5G). Therefore, improved killing in CT2A and astrocyte co-cultures is not due to astrocyte cell death-related paracrine signaling on SMC-sensitized GBM cells, but instead likely because of additional secreted factors from the astrocytes themselves.

Inflammatory astrocyte reactive states are characterized by increased inflammatory cytokine production [62]. Proinflammatory cytokines that activate NF-κB, such as IL-1β, result in production of TNF-α and consequently can lead to autocrine and paracrine cell death signaling to SMC-sensitized cells [63]. Notably, TNF-α expression from activated microglia is a known inducer of astrocyte reactive states [64]. Understanding the impact of IAP inhibition on astrocytes is key, as this can impact on microglia as well [65]. We evaluated whether SMCs can alter astrocyte phenotypes in a co-culture setting. Consistent with the findings of Fig. 5E, the presence of astrocytes buffered the growth inhibitory effects of repeat high-dose SMC treatment (Fig. 5H). High dose LCL161 increased expression of astrocyte reactivity markers GFAP and C3c but not PD-L1 (Fig. 5I). Although LCL161 can mildly stimulate GFAP reactivity, the amounts were not comparable to classical inducers such as LPS and TNF-α (Fig. 5J). Given these, it appears as though TNF-α is the major inducer of astrocyte reactive states, which would then promote the death of SMC-sensitized GBM cells.

### Astrocytes protect microglia and GBM cells from the cytotoxic effects of high dose SMCs

**Fig. 6:**
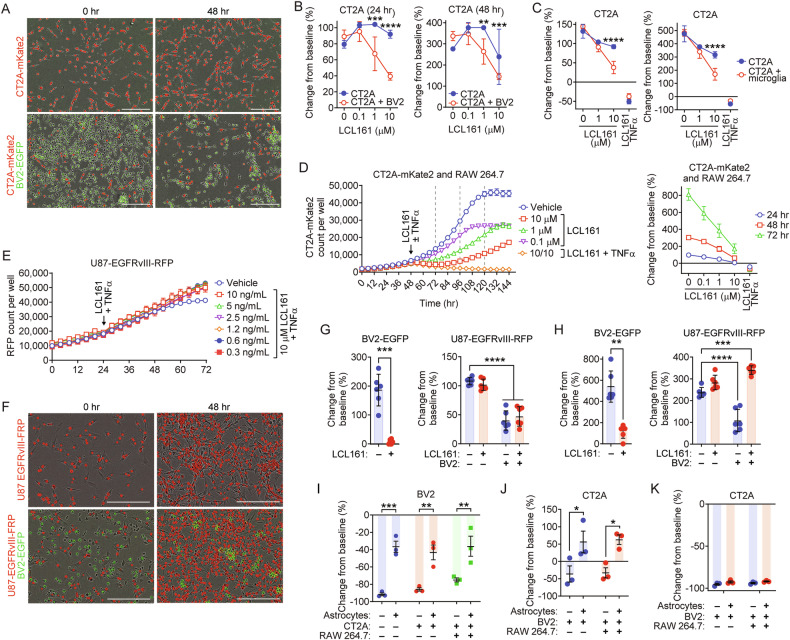
Presence of microglia and macrophages confers monotherapeutic efficacy to LCL161 in SMC-sensitive GBM killing, with both cell types protected by astrocytes. **A**, **B** BV2-EGFP cells were cultured 1:1 with CT2A-mKate2 cells and treated 24 h later with tenfold dilution series of LCL161. Viable cells were enumerated by tracking mKate2-positive events using live cell imaging over 48 h post treatment. *N* = 3 for monocultures, *N* = 6 for co-cultures per treatment group. Scale bar: 300 µm. **C** CT2A-mKate2 cell numbers were analyzed and treated as in (**B**) in co-culture with primary mouse microglia over 48 h. Response to treatment with 10 µM LCL161 and 10 ng/mL TNF-α was also assessed. *N* = 4 per treatment group. **D** CT2A-mKate cells were cultured with RAW macrophages and treated with indicated doses of LCL161 and 10 ng/mL TNF-α. Cell numbers were assessed as in (**B**). Percent change over 72 h was calculated from bulk mKate2-positive events (left) and plotted (right). The arrow indicates treatment point, dashed lines indicate time points for subsequent calculations. *N* = 3 per treatment group. **E** U87-EGFRvIII-RFP human GBM cells were treated with indicated dilution series of TNF-α in the presence of 10 µM LCL161. The arrow indicates treatment point. **F**–**H** U87-EGFRvIII-RFP cells were co-cultured 1:1 with BV2-EGFP cells and treated 24 h later with 10 µM LCL161. Viable cells were enumerated by tracking RFP- and EGFP-positive events using live cell imaging 24 h (**G**) and 48 h (**H**) post treatment. *N* = 3 per treatment group. Scale bar: 300 µm. **I**, **J** BV2-EGFP and CT2A-mKate2 counts in co-cultures with RAW cells and primary mouse astrocytes treated with 10 µM LCL161 for 72 h. EGFP and mKate2-positive events were assessed using live cell imaging. *N* = 3 per treatment group. **K** Co-cultures in (**I**, **J**) treated with combination 10 µM LCL161 and 10 ng/mL TNF-α for 72 h and analyzed as before. *N* = 3 per treatment group. *P < 0.05; **P < 0.01; ***P < 0.001; ****P < 0.0001 by two-way ANOVA using Tukey’s HSD multiple comparison test.

To assess whether interactions between microglia, macrophages, astrocytes and GBM cells affect sensitivity to SMC-mediated death of each cell type, a series of co-culture experiments were undertaken. Consistent with previous findings, BV2 microglia remained sensitive to high dose LCL161 in co-culture with CT2A cells. Loss of BV2 viability translated to reduced growth of CT2A cells relative to monocultures (Fig. 6A, B), suggesting that the presence of microglia can transform LCL161 into a death signal for GBM cells independent of the addition of exogenous TNF-α. We observed a similar trend with primary microglia over 24 and 48 h (Fig. 6C). The presence of microglia or BMDMs (Supplementary Fig. 8) had no impact on sensitivity of CT2A cells to combined LCL161 and TNF-α treatment, suggesting that the cell death pathways are still intact in these co-cultures. Similarly, we observed the same reduced CT2A growth rate in co-cultures with RAW macrophages, both with LCL161 alone or combination with TNF-α (Fig. 6D). Lower doses of LCL161 also caused significant reductions in growth, likely a result of the greater sensitivity of RAW cells to LCL161-induced cell death compared to BV2 (Fig. 3A–C). To determine whether this is unique to SMC-sensitive GBM cells or whether SMC-treated microglia in these co-cultures can induce cell death of SMC-resistant GBM, RFP-tagged U87 human GBM cells expressing EGFRvIII, which are resistant to LCL161 and TNF-α treatment (Fig. 6E, Supplementary Fig. 9), were co-cultured with BV2 mouse microglia and treated with 10 µM LCL161. BV2-EGFP and U87-RFP cell numbers were assessed in response to SMC treatment over the subsequent 48 h. BV2 numbers were decreased by SMC treatment while U87 cells were unaffected. Loss of microglia from co-culture enhanced U87 human GBM growth (Fig. 6F–H, Supplementary Fig. 10). Therefore, SMC-induced death of microglia and macrophages releases factors that can subsequently kill SMC-sensitive but not resistant GBM cells.

We next assessed the impact of SMCs on cell viability in triple and quadruple co-culture conditions. LCL161 treatment results in a near complete loss of BV2 cells within 72 hr; the addition of CT2A or RAW cells had no significant impact on this effect (Fig. 6I). However, regardless of the complexity of co-culture, the presence of astrocyte -protected microglia from the cytotoxic effects of LCL161. As the LCL161-induced death of microglia has been shown to be cytotoxic to CT2A cells under co-culture conditions (Fig. 6A–C), we next aimed to determine whether this astrocyte buffering of LCL161 cytotoxicity translated to protection of CT2A cells. LCL161 (10 µM) treatment reduced CT2A numbers when co-cultured with BV2 and RAW cells; the presence of astrocytes increased growth from baseline (Fig. 6J), illustrating a protective effect of astrocytes on microglia and GBM cells in vitro. As astrocytes enhance the cell death of CT2A cells when TNF-α is added in combination with LCL161 (Fig. 5F), we aimed to determine whether the presence of macrophages or microglia impacted this sensitivity. A near-complete loss of CT2A cells was seen following combination LCL161 (10 µM) and TNF-α (10 ng/mL) treatment regardless of populations present (Fig. 6K).

## Discussion

GBM represents one of the deadliest and most expensive cancers to treat. The location of GBM within the brain limits drug delivery and anti-tumor immunity. Moreover, high intra- and inter-tumoral heterogeneity and significant myeloid immunosuppression add to the difficulty in developing effective treatments. Recent research has highlighted a central role of the IAP family member cIAP2 in the genesis and lethality of GBM [10, 12, 13, 66]. We show that under half of commonly used human GBM lines are sensitive to SMC-induced inflammatory cell death. While all commonly used human GBM lines express full extrinsic apoptotic machinery, resistance to cell death is achieved through the actions of cellular FLICE-like inhibitory protein (cFLIP). Differential regulation of cFLIP degradation via JNK-ITCH signaling confers differential sensitivities to SMC-mediated cell death [16]. Structural similarity of cFLIP to procaspase-8 limit its pharmacological targetability, however recent work has shown encouraging results in designing cFLIP-specific compounds with the potential to substantially improve SMC-efficacy [67]. Along with their role in enhancing many aspects of the cancer-immunity cycle, SMC targeting of the cIAPs has significant benefit for the treatment of this highly resistant cancer. In this report, we assessed the importance of stromal components in the efficacy of SMC-mediated GBM cell death. Here, we show that murine GBM cells do not acquire cell intrinsic resistance mechanisms to SMC treatment. Instead, insufficient neuroinflammation, SMC drug delivery to tumor and astrocyte protection represent likely modes of resistance to SMC therapies.

Astrocytes have been demonstrated to provide protective effects against chemotherapies [59, 68], a feature consistent with our findings. In vitro SMC treatment led to increased expression of GFAP and C3c in astrocytes, which can in turn lead to expression of other factors that enhance GBM growth, such as TGF-β, IL-6, and IGF-1 [69, 70]. Even at high doses of SMC treatment this effect is insufficient to affect GBM cell growth in astrocyte-GBM co-culture. SMCs do not affect astrocyte viability even in the presence of exogenous TNF-α, consistent with well-characterized resistance to extrinsic apoptotic triggers [71, 72]. However, the inclusion of TNF-α with SMCs in astrocyte-GBM co-cultures led to increased cytotoxicity towards GBM cells. TNF-α is a known inducer of astrocyte reactivity [64], which can in turn increase expression and secretion of TNF-α, acting on sensitized GBM cells to enhance cytotoxicity of SMCs. In vivo SMC treatment increases astrocyte involvement surrounding tumor, which may provide death ligands for potentiating SMC-mediated GBM killing or limiting GBM invasive potential [73]. Thus, SMCs can induce inflammation and induce astrocyte reactivity, however, this itself is insufficient for astrocytes to secrete high enough levels of TNF-α that lead to GBM cell death.

The therapeutic efficacy of SMCs can also be related to high dose of SMCs to induce death of microglia and macrophages. In co-culture conditions, this microglia/macrophage death acts in a paracrine fashion to kill sensitive GBM cells, conferring LCL161 monotherapeutic efficacy in the absence of exogenous TNF-α. The M1 proinflammatory subset of microglia and macrophages are relatively more sensitive to SMC-induced death, consistent with past findings in SMC sensitivity in human macrophages [74] and noted enhanced sensitivity to apoptosis in cIAP2 deficient murine macrophages [75]. The addition of astrocytes protects macrophages, microglia and GBM cells from this cytotoxicity, implicating a role for astrocytes promoting an immunosuppressive environment that inhibits SMC-mediated death of GBM cells. We show that this effect is limited to SMC-sensitive GBM cells, as the resistant U87 line shows no reductions in viability under the same co-culture conditions. Nonetheless, microglia were susceptible to SMC-mediated cell death, and even in the presence of SMC-resistant GBM cells the ability of SMCs to reduce immunosuppressive TAMM populations, increase inflammatory cytokine release and cumulatively promote astrocyte reactivity surrounding tumor can significantly enhance any immunotherapy.

The use of SMCs for treatment of GBM is a promising approach, especially as cIAP2 is a known oncogene for GBM tumorigenesis. We have shown here that SMC resistance is not due to sustained cell-intrinsic mechanisms within the brain parenchyma. In addition, TME factors, such as hypoxia, hypoglycemia, and interactions with the brain ECM, do not have a significant role in SMC resistance. Rather we found that the therapeutic efficacy is enhanced with SMC-induced death of TAMMs but is countervailed by astrocytes. This resistance is overcome with high levels of TNF-α, of which this cytokine can be induced through inflammatory approaches, such as immunostimulants [40, 44, 63]. However, it remains to be seen whether localized or systemic delivery of SMCs is more efficacious either as a monotherapy or in combination with other therapies. For example, Temozolomide administered systemically reduces the efficacy of the immune checkpoint inhibitor α-PD-1, with potent immune toxicity; conversely, intratumoral delivery significantly enhances anti-tumor immune responses [76, 77]. Exploration of more direct treatment applications such as intratumoral or intracerebroventricular delivery of SMC, or slow release wafers implanted in surgical bed as used for carmustine treatment of high-grade gliomas [78] represent key future research areas to maximize SMC-mediated GBM killing and neuroinflammatory responses. In murine models, dose escalation of SMCs as well as combination with TGFβ blockade represent key future research areas, both looking to enhance SMC effects on TAMMs and consequent engagement of neuroinflammatory processes. This two-pronged attack of increasing peripheral inflammation by inducing cell death of immunosuppressive populations within the tumor, while engaging neuroinflammatory astrocytes, act together to sensitize GBM cells to SMC-induced death and represents a promising path for treating GBM.

## Methods

### Reagents

LCL161 was purchased from SelleckChem (Houston, TX, USA) or provided by Novartis (Basel, Switzerland).AZD-5582 (A-9051) was purchased from Active Biochem. Mouse TNF-α (410-MT-010), TGF-β (7666-MB-005) and IFNγ (4875-M1) were purchased from R&D systems (Minneapolis, MN, USA). Murine IL-4 (214-14) was purchased from Peprotech (Cranbury, NJ, USA). Mouse IL-10 (575806) was purchased from BioLegend (San Diego, CA, USA). LPS (tlrl-peklps) was purchased from Invivogen (San Diego, CA, USA). Z-VAD-FMK (FMK001) was purchased from R&D systems. Necrostatin-1 (N9037) was purchased from Sigma Aldrich (St. Louis, Missouri, USA).

### Cell culture

Cells were maintained at 37 °C and 5% CO_2_ in DMEM media supplemented with 10% heat-inactivated fetal calf serum, 1% non-essential amino acids, and penicillin-streptomycin (Invitrogen). Cell lines were obtained from ATCC, with the following exceptions: BV2 (Dr. Shawn Hayley, Carleton University); RFP-tagged U87-EGFRvIII (Dr. Scott McComb, NRC Canada); and SMA-560 (A-9051, EMD Millipore (Burlington, MA, USA)). Cells were regularly tested for mycoplasma. BV2-GFP cells were generated using IncuCyte NucLight Green reagent (4626) from Sartorius/Essen Bioscience (Ann Arbor, MI, USA). For hypoxia experiments, oxygen levels were altered using ProOx 110 Compact O_2_ Controller ((RRID: SCR_021129) from Biospherix, Ltd (Parish, NY, USA). No glucose conditions were achieved using DMEM complete media without glucose (11966025) from Thermo Fisher (Waltham, MA, USA). Mouse primary cortical astrocytes (M1800-57) and microglia (M1900-57) were purchased from ScienCell (Carlsbad, CA, USA). Astrocytes were cultured in astrocyte media from ScienCell (1801), supplemented with 2% FCS, 1% penicillin/streptomycin and 1% astrocyte growth serum (ScienCell). For culture on ECM proteins, cells were seeded on culture plates coated with HyStem-C cell culture scaffold kit (HYSC020) from Sigma Aldrich. For brain slice cultures, slices were generated and maintained as previously described. Briefly, female 5–7-week-old C57BL/6 mice were sacrificed and their cortices isolated. Forebrain was fixed in ultra-low melting point agarose. Embedded brain was cut on ice into 250 μm slices using a Leica Biosystems (Wetzlar, Germany) VT1000 S vibratome. Slices were placed in a 0.4 μm Millicell cell culture insert from Sigma (PICM0RG50), which was in turn placed in a 6-well plate overtop NeuroCult Neural Stem Cell media (05700), supplemented with recombinant basic FGF (bFGF, 78003) and EGF (78006) from StemCell (Vancouver, Canada). Two days later slices were imaged and visually assessed for viability or contamination; healthy slices were kept and the rest discarded. Following this, an indentation was made in the left striatum using a 10 μL pipette tip and 5 × 10^3^ CT2A-mKate cells were implanted in and around the indentation pit. The following day, adherence of cells on brain tissue was confirmed using an EVOS fluorescent microscope. Media was changed every 2–3 days. After five days of growth, whole slices were imaged using an EVOS fluorescent microscope to give a baseline CT2A-mKate count. Following this, either fresh media, 1 μM LCL161 + 1 ng/mL TNF-α or 10 μM LCL161 + 10 ng/mL TNF-α was added to the culture media below the culture insert. After 24 h, slices were again imaged using the EVOS fluorescent microscope. Surviving red CT2A-mKate nuclei were manually counted using ImageJ, and the percent change from baseline prior to treatment was calculated. For spheroid cultures, 400 cells/well of CT2A or GL261 cells were plated on Corning (Corning, New York, USA) Costar ultra-low attachment well plates (CLS7007) in NeuroCult Neural Stem Cell media supplemented with bFGF and EGF. Growth was tracked using Incucyte live cell analysis system.

### In vitro viability assay

Cell lines were seeded at 1 × 10^4^ cells/well in 96-well plates and incubated overnight. Cells were treated with 50% dilution series of LCL161, TNF-α or the combination or matched DMSO and media control for 24, 48 or 72 h. Viability was assessed using Alamar blue (resazurin sodium salt (Sigma)), with treated cell readouts normalized to matched vehicle control.

### Western blotting

Cells were scraped, collected by centrifugation and lysed in RIPA lysis buffer containing protease inhibitor cocktail (Roche (Basel, Switzerland)). Equal amounts of soluble protein were separated on polyacrylamide gels followed by transfer to nitrocellulose membranes. Individual proteins were detected using antibodies for: cIAP1/2 (1:1000, CY-P1041, Cyclex); XIAP (1:1000, 2042S, Cell Signaling (MA, USA)); Caspase-8 (1:1000, R&D); Caspase-3 (1:1000, 9662S, Cell Signaling); and PARP (1:1000, P532S, Cell Signaling). Full blots (Supplementary Fig. 7) were cropped as indicated (red boxes) and BV2 bands mirrored horizontally so the order of treatments was consistent with the RAW264.7 data (Fig. 3J).

### Flow cytometry

For chronic LCL161 treatments, GL261 and CT2A cells were treated daily for 10 days with 10 μM LCL161 or matched DMSO. Cells were lifted from plates using non-enzymatic cell dissociation buffer (C5914) from Sigma and stained for viability with Zombie Violet fixable viability kit (423114, BioLegend) and Fc block (101320, BioLegend) at 1:300 dilution and subsequently stained for CD44 expression (PE conjugate, 1:200, 12-0441-82 from eBioscience). For astrocyte reactivity marker expression, cells were treated for 24 h with 10 μM LCL161 or matched DMSO and lifted as described previously. Cells were stained for viability as before, and subsequently stained for PDL1 (APC conjugate, 1:200, 124311, BioLegend) and C3c (FITC conjugate, 1:200, ab4212, Abcam). Following surface stains, cells were permeabilized using eBioscience intracellular fixation and permeabilization buffers (88-8824-00) and stained for GFAP (AlexaFluor594 conjugate, 1:200, 644708, BioLegend).

### Microscopy

Detection of CT2A-mKate2 and BV2-GFP cells were performed in an incubator outfitted with an Incucyte Zoom microscope under 10× objective. For assessment of cleaved caspase-3, Incucyte Caspase-3/7 green dye for apoptosis from Essen Bioscience (4440) was used. Quantification of fluorescent signal was processed using Incucyte Zoom software. For brain slice cultures, phase and fluorescent images were acquired using EVOS FL Auto on stitched 20x objective images. For astrocyte GFAP fluorescence, mouse cortical astrocytes were grown in ibidi microslide 8-well-chambered coverslips (80826) and treated for 24 h with DMSO, 10 μM LCL161, 500 ng/mL LPS or 10 ng/mL TNFα. Cells were then stained in-well for GFAP expression (ab10062, Abcam, 1:100) and images were acquired on Zeiss (Baden-Wurttemberg, Germany) Axio Imager.M2 at 20x objective. For tumor-bearing whole brain stains, mice were transcardially perfused on day 18 post-implant and formalin-fixed in 4% paraformaldehyde (PFA) for 48 h. Fixed brains were sent to the Louise Pelletier Histology Core at the University of Ottawa for paraffin embedding and slicing (4 μm thick slices). Slices were stained using the Akoya (Marlborough, MA, USA) Opal polaris 7-color IHC detection kit (NEL811001KT) using protocol outlined by Allam and Russell, 2022 [79]. Background autofluorescence was quenched using TrueBlack lipofuscin autofluorescence quencher (1:20, 10119-144, Biotium (Fremont, CA, USA)). Brains were stained with DAPI (0.5 μg/mL, PerkinElmer (Waltham, MA, USA)), anti-mouse GFAP (1:100, MA5-12023, Life Technologies (Carlsbad, CA, USA)) following pH9 antigen retrieval, Opal Polymer HRP Ms+Rb (ARH1001EA, Akoya Biosciences) and signal generated using Opal 570 dye (1:100 in 1× Plus amplification diluent, FP1609, Akoya). Slides were mounted and IHC images acquired using Zeiss AxioImager Z2 widefield microscope with Colibri 5/7 and Apotome at 10x. Images were stitched in Zeiss Zen Black software. Analysis was performed using ImageJ.

### CT2A and GL261 brain tumor models

Female 6-week-old C57BL/6 mice were anesthetized with isofluorane and the surgical site was prepared. 5 × 10^4^ cells were implanted stereotactically over 1 min in a 10 μL volume in the left striatum at coordinates: 0.5 mm anterior, 2 mm lateral from bregma, 3.5 mm deep. Skin was closed using surgical glue. Mice were treated with either vehicle (30% 0.1 M HCl, 70% 0.1 M NaOAc pH 4.63) or 75 mg/kg LCL161 resuspended in 30% 0.1 N HCl and 70% CH_3_COONa. For treatment with checkpoint inhibitors (10 mg/kg), mice were treated with anti-PD-1 clones J43 (BE0033-2), 29 F.1A12 (BE0273) or RMP1-14 (BE0146) or IgG2A isotype control (BE0089 or BE0091, where applicable) from BioXcell (Lebanon, NH, USA). Animal endpoint criteria include loss of >20% body weight, hunched posture, lethargy and significantly impaired ambulation. For isolation of endpoint tumor cells, animals at endpoint were sacrificed, tumor bulk dissected from brain, and CT2A or GL261 cells isolated using Tumor Cell Isolation Kit (130-110-187) from Miltenyi Biotec (North Rhine-Westphalia, Germany). Experimental groups were not blinded and no randomization of treatment groups were performed. The sample size is consistent with previous reports but no statistical methods were used to determine sample size [37, 40, 44].

### Ethics statement

All animal experiments were conducted with the approval of the University of Ottawa Animal Care and Veterinary Service in concordance with guidelines established by the Canadian Council on Animal Care (CHEO-3163).

### Statistical analysis

Comparison of Kaplan–Meier survival plots was conducted by long-rank analysis and subsequent pairwise multiple comparisons were performed using the Holm-Sidak method (GraphPad). Comparison between multiple treatment groups was analyzed using one- or two-way ANOVA followed by post hoc analysis using Tukey’s multiple comparison test (GraphPad). Comparison of treatment pairs was analyzed by two-sided t-tests (GraphPad). Area under the curve was calculated using GraphPad. Estimate of variation was analyzed with GraphPad. Lines within bar graphs represent mean with standard error.

### Supplementary information

Supplemental Figures

Original Data (Western blots)

## Data Availability

The datasets generated during and/or analyzed during the current study are available from the corresponding author on reasonable request.
