# Supplementary material for: Astrocytes and the tumor microenvironment inflammatory state dictate the killing of glioblastoma cells by Smac mimetic compounds
Source: Cell Death Dis. 2024 Aug 15;15(8):592. doi: 10.1038/s41419-024-06971-5 (PMC11327263; doi:10.1038/s41419-024-06971-5)
Supplement: Supplementary file 1 — Supplemental Figures [file 41419_2024_6971_MOESM1_ESM.pdf]

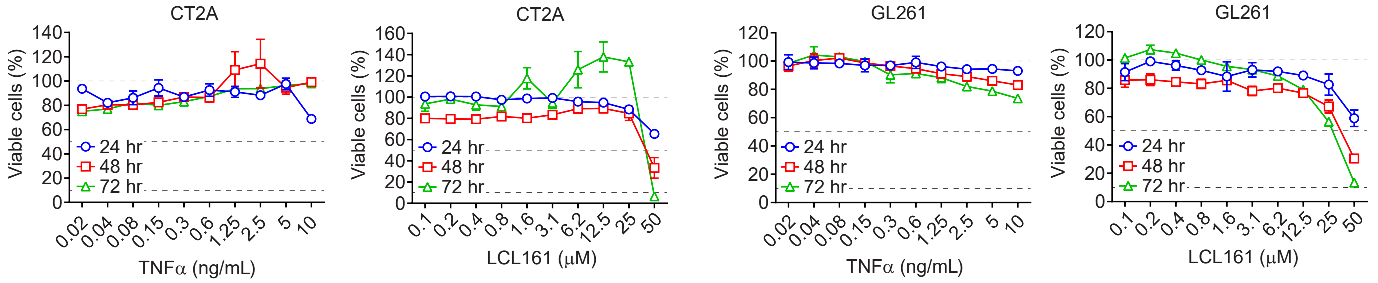

Supplementary Figure 1: Murine GBM cells are refractory to death induced by single agent treatment with SMC or TNF-α. CT2A and GL261 cells were treated for 72 hr with the indicated concentrations of LCL161 or TNF-α. Viability was assessed using Alamar blue. N=3 per treatment group.

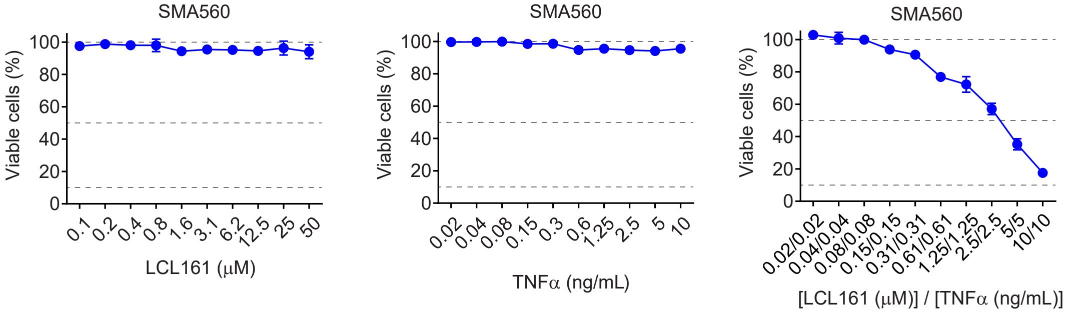

Supplementary Figure 2: SMA-560 murine GBM cells are sensitive to combination SMC and TNF-α. SMA-560 cells were treated for 24 hr with the indicated concentrations of LCL161, TNF-α or the combination. Viability was assessed using Alamar blue. N=3 per treatment group.

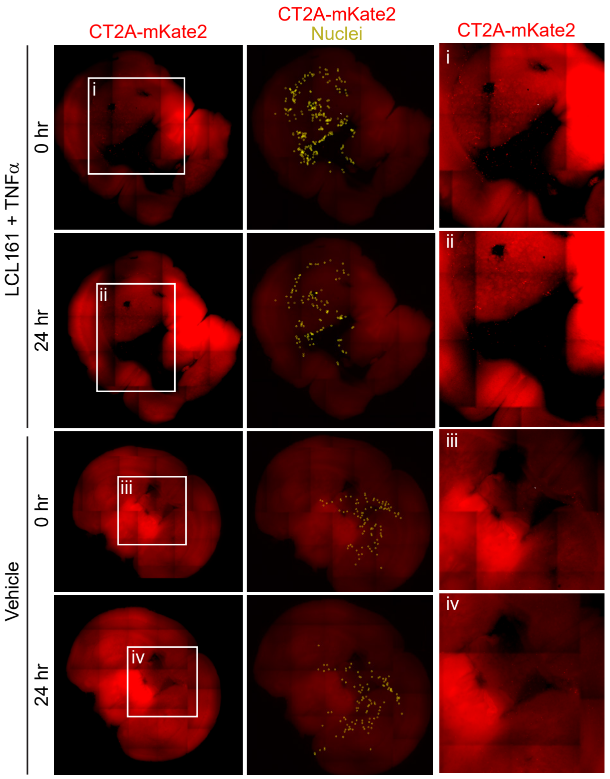

Supplementary Figure 3: Representative images of SMC-treated organotypic brain cultures implanted with murine GBM cells. CT2A-mKate cells were cultured on 250 μm thick mouse brain slices. mKate2 positive events were identified and overlaid on top of the images. Cells growing in the ventricle were excluded from analysis.

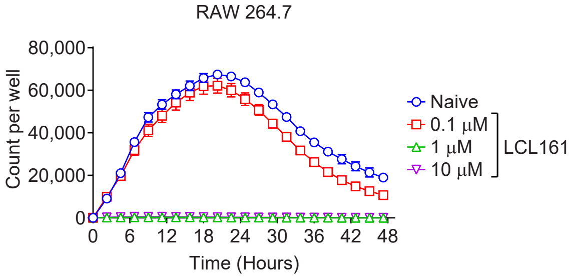

Supplementary Figure 4: SMC treatment attenuates phagocytic capacity of immortalized murine macrophages and microglia. RAW 264.7 and BV2 were assayed in the presence of the indicated concentrations of LCL161 for phagocytic activity of pHrodo bioparticles via live cell microscopy. N=6 per treatment group.

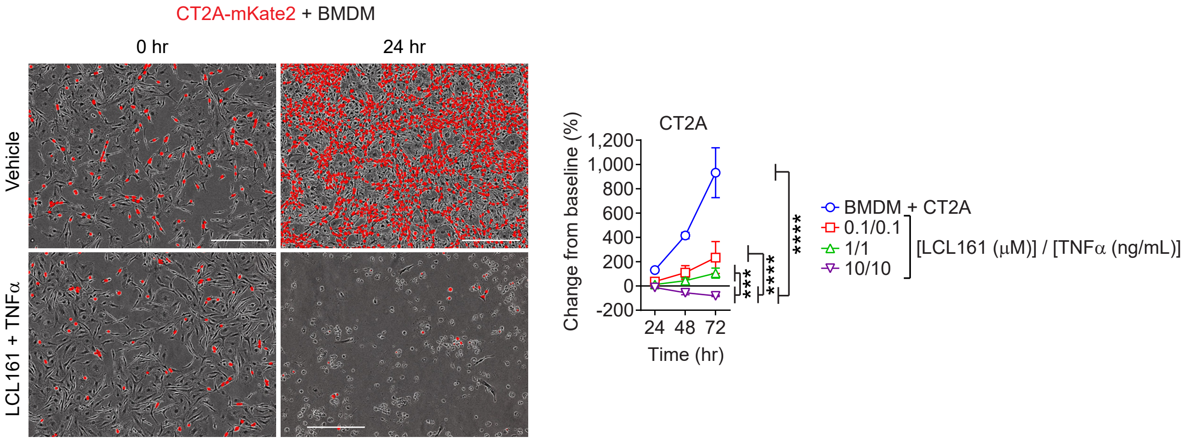

Supplementary Figure 5: BV2 microglia remain sensitive to LCL161 regardless of polarization in FCS-free conditions. BV2-EGFP cells were pretreated with LPS (100 ng/mL), IL-4 or IL-10 (20 ng/mL) for 24 hr in FCS-free conditions. Cells were then treated with the indicated LCL161 concentrations or with 10 μM LCL161 and 10 ng/mL TNF-α. BV2-EGFP cell numbers were counted via live microscope imaging. N=3 per treatment group.

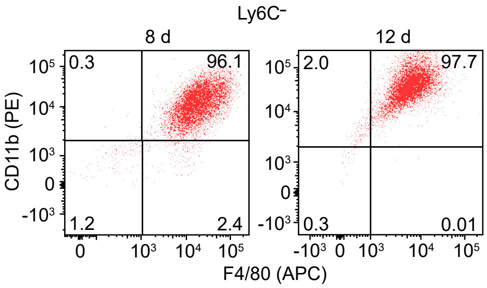

Supplementary Figure 6: Confirmation of BMDM differentiation. Bone marrow cells were differentiated into macrophages using L929 conditioned media for 8 or 12 days. Cells were then evaluated for the expression of Ly6C, CD11b and F4/80 via flow cytometry.

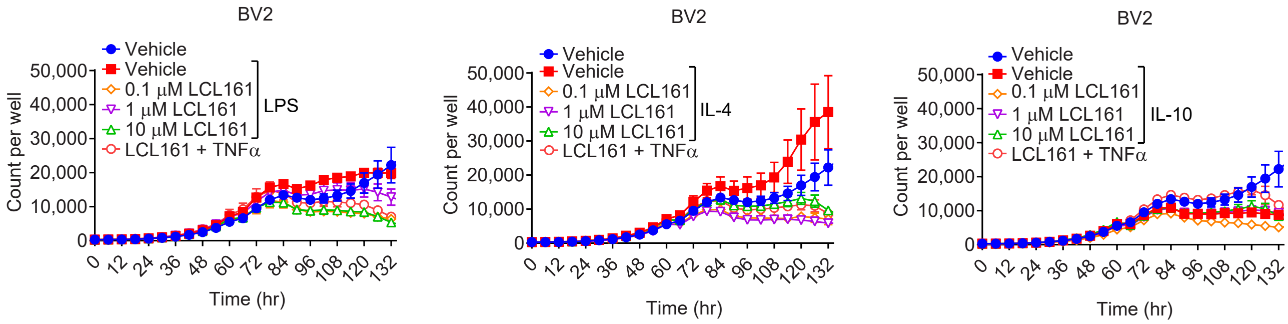

Supplementary Figure 7: CT2A-mKate2 growth is increased by the presence of astrocytes. Growth curves of CT2A-mKate2 cells cultured alone or with mouse astrocytes and treated with indicated concentrations of LCL161 and TNF-α. Data points are plotted as percent change from initial seeding count. Dashed lines were used to calculate data plotted in Figure 5E.

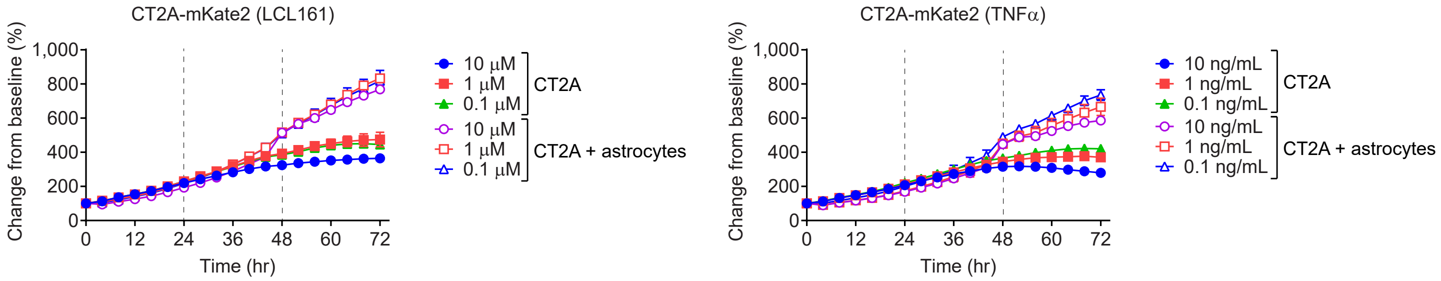

Supplementary Figure 8: Murine GBM cells remain sensitive to LCL161 and TNF-α treatment when co-cultured with BMDMs. CT2AmKate cells were co-cultured with BMDMs and treated with indicated doses of LCL161 and TNF-α. CT2A-mKate2 cells were enumerated via live microscope imaging of mKate2 events. N=3 per treatment group.

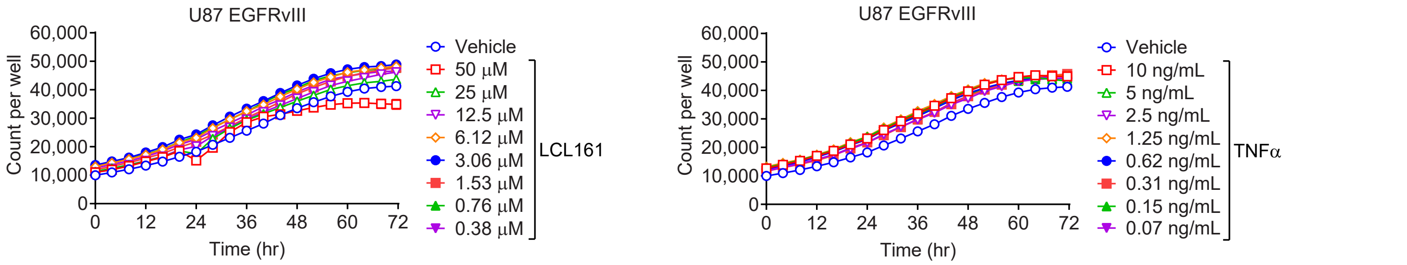

Supplementary Figure 9: Human U87-EGFRvIII GBM cells are refractory to death induced by single agent treatment with SMC or TNF-α. U87-EGFRvIII-RFP GBM cells were treated with indicated concentrations of LCL161 or TNF-α (arrow) and RFP count enumerated over the subsequent 48 hr using live cell imaging. N=3 per treatment group.

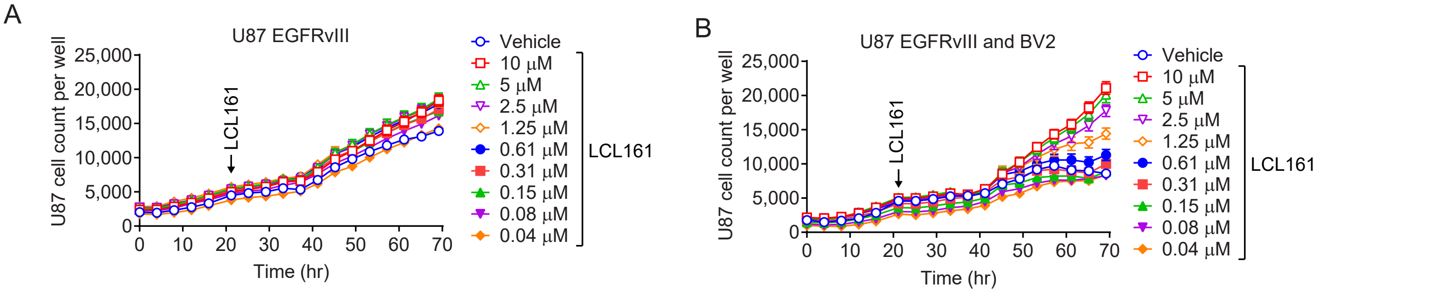

Supplementary Figure 10: U87 human GBM cells remain resistant to LCL161 in the presence of dying microglia. U87-EGFRvIII-RFP cells cultured alone (left) or with BV2-EGFP cells (right) and treated with indicated concentrations of LCL161. U87-EGFRvIII-RFP cells were enumerated using live microscope imaging of RFP events. N=3 per treatment group.
