# Supplementary figures and images for: Astrocytes and the tumor microenvironment inflammatory state dictate the killing of glioblastoma cells by Smac mimetic compounds
Source: Cell Death Dis. 2024 Aug 15;15(8):592. doi: 10.1038/s41419-024-06971-5 (PMC11327263; doi:10.1038/s41419-024-06971-5)

Figure 3J

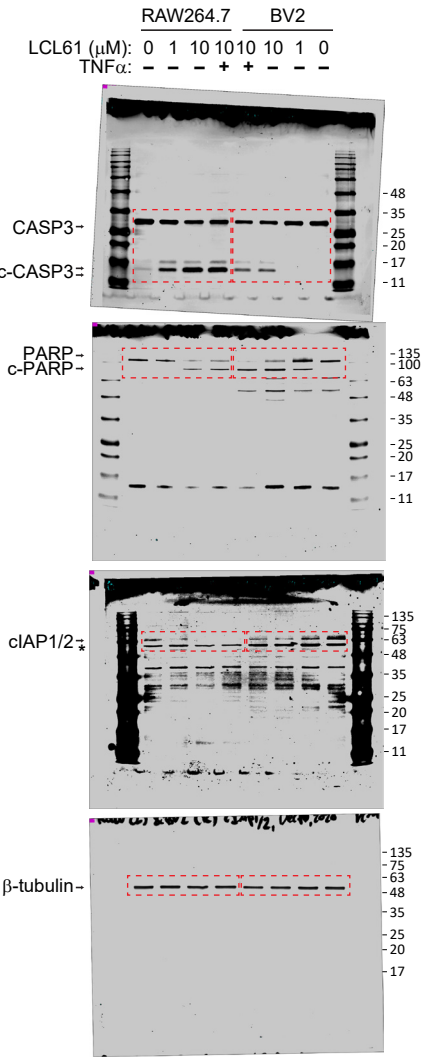

Supplement: Supplementary file 2 — Original Data (Western blots) [file 41419_2024_6971_MOESM2_ESM.pdf]
